# Inhibition of Fatty Acid Amide Hydrolase (FAAH) Regulates NF-kb Pathways Reducing Bleomycin-Induced Chronic Lung Inflammation and Pulmonary Fibrosis

**DOI:** 10.3390/ijms241210125

**Published:** 2023-06-14

**Authors:** Tiziana Genovese, Andrea Duranti, Francesco Monaco, Rosalba Siracusa, Roberta Fusco, Daniela Impellizzeri, Ramona D’Amico, Marika Cordaro, Salvatore Cuzzocrea, Rosanna Di Paola

**Affiliations:** 1 Department of Chemical, Biological, Pharmaceutical and Environmental Sciences, University of Messina, Viale Ferdinando Stagno D’Alcontres 31, 98166 Messina, Italyrsiracusa@unime.it (R.S.); dimpellizzeri@unime.it (D.I.);; 2 Department of Biomolecular Sciences, University of Urbino Carlo Bo, Piazza del Rinascimento, 6, 61029 Urbino, Italy; 3 Department of Biomedical and Dental Sciences and Morphofunctional Imaging, University of Messina, 98166 Messina, Italy; 4 Department of Veterinary Sciences, University of Messina, 98168 Messina, Italy; dipaolar@unime.it

**Keywords:** chronic lung injury, bleomycin, inflammation, fatty acid amide hydrolase

## Abstract

The deadly interstitial lung condition known as idiopathic pulmonary fibrosis (IPF) worsens over time and for no apparent reason. The traditional therapy approaches for IPF, which include corticosteroids and immunomodulatory drugs, are often ineffective and can have noticeable side effects. The endocannabinoids are hydrolyzed by a membrane protein called fatty acid amide hydrolase (FAAH). Increasing endogenous levels of endocannabinoid by pharmacologically inhibiting FAAH results in numerous analgesic advantages in a variety of experimental models for pre-clinical pain and inflammation. In our study, we mimicked IPF by administering intratracheal bleomycin, and we administered oral URB878 at a dose of 5 mg/kg. The histological changes, cell infiltration, pro-inflammatory cytokine production, inflammation, and nitrosative stress caused by bleomycin were all reduced by URB878. Our data clearly demonstrate for the first time that the inhibition of FAAH activity was able to counteract not only the histological alteration bleomycin-induced but also the cascade of related inflammatory events.

## 1. Introduction

Idiopathic pulmonary fibrosis (IPF) is a form of interstitial lung disease that progresses and ultimately ends in death. Its cause is unknown. IPF resembles the histopathological pattern of typical interstitial pneumonitis and is characterized by areas of peripheral fibrosis, interstitial inflammation, failure of alveolar re-epithelialization, persistence of fibroblasts/myofibroblasts, and deposition of extracellular matrix (ECM) molecules in the lung [1]. Respiratory failure and lung architecture distortion may be the last effects of IPF progression, and the estimated 5-year survival rate for IPF is 20% [2].

The pathophysiology of interstitial lung disease includes inflammation, which is partly regulated by endogenous and migratory leukocytes. These leukocytes create a feedback loop where inputs from damage responses can activate alveolar and interstitial macrophages, along with lung epithelial and endothelial cells [3].

Additionally, inflammatory reactions are linked to the early stages of IPF, and nuclear factor kappa B (Nf-kb), a transcription factor, is essential for controlling several genes that produce cytokines that are both pro-inflammatory and pro-fibrogenic. Tumor necrosis factor-α (TNF-α) has been found to be a powerful inflammatory cytokine that is produced during the inflammatory stage of wound healing and has been linked to the development of IPF. TNF- may boost the expression of transforming growth factor β (TGF-β) and expedite the epithelial to mesenchymal transition (EMT) in mice primary lung fibroblasts [4]. Thanks to the histological similarities between bleomycin-treated animals and people with idiopathic pulmonary fibrosis, such as mural incorporation of collagen, intra-alveolar buds, and obliteration of the alveolar spaces, the bleomycin animal model is typically used to assess the in vivo efficacy of antifibrotic agents [5]. Intratracheal instillation of bleomycin produces an early inflammatory reaction marked by the overexpression of proinflammatory cytokines such as interleukin-6 (IL-6), interleukin-1β (IL-1β), and tumor necrosis factor-α (TNF-α), followed by increased levels of profibrotic markers, such as transforming growth factor-β1 (TGF-β1), fibronectin, and procollagen-1 [6]. By day nine following bleomycin instillation, the inflammatory and fibrotic stages “flip” over to one another [6]. As a result, medications given during the first seven days may primarily operate as anti-inflammatory medicines and are referred to as “preventive or prophylactic”, whereas medications given between days seven and ten may be real antifibrotic medications and are referred to as “therapeutic” [6].

Fatty acid amide hydrolase (FAAH) is an enzyme that catalyzes the hydrolytic metabolism of important natural amides, such as mainly N-arachidonoylethanolamide (AEA) but also palmitoylethanolamide (PEA), N-oleoylethanolamide (OEA), and linoleoylethanolamide (LEA), with a consequent reduction of their half-life. These ligands are involved in various processes so their right concentrations are important for the regulation of physiological balances. Proof of this are the advantages deriving from the administration of FAAH inhibitors from in vivo experimental models, also carried out with knockout animals [7,8,9,10,11,12,13]. As an example, the pharmacological tool URB597, which has demonstrated benefits in a large number of disorders, whether they are central or peripheral (e.g., anxiety, depression, analgesia, neuropathic pain, inflammation) without the undesirable effects deriving from the direct activation of cannabinoid receptors [7,8,9,10,11,12,13,14,15,16,17,18,19,20,21,22,23,24,25,26]. In particular, great interest has been seen in the field of inflammation [27,28].

Given such a scenario, it is very interesting to investigate the role played by FAAH through the very potent inhibitor URB878 (4-phenylbutylcarbamic acid 3′-carbamoylbiphenyl-3-yl ester) [29,30,31].

The aim of this study was to investigate for the first time the role of FAAH inhibition in chronic lung injury, bleomycin-induced. To do this, a new pharmacological tool was used such as the FAAH inhibitor URB878.

## 2. Results

### 2.1. URB878 Reduced Bleomycin-Induced Mortality, Body Weight Decreases, and Histological Damage

**Figure 1:**
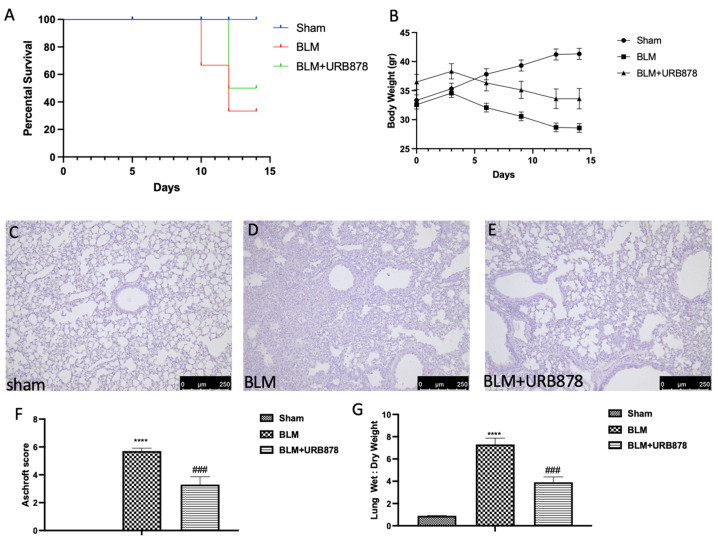
URB878 reduced bleomycin-induced mortality, body weight decreases, and histological damage. Mortality (**A**), and body weight (**B**). Histological photo of: (**C**) sham, (**D**) bleomycin, (**E**) sbleomycin+URB878 5 mg/kg; (**F**) Ashcroft score; (**G**) wet:dry lung weight ratio. The data are expressed as the mean ± SEM of n = 6 animals for each group. **** *p* < 0.0001 vs. sham; ### *p* < 0.001 vs. bleomycin.

URB878 was given daily for a total of 14 days in order to assess the possibility of FAAH inhibition for the treatment of fibrosis. Death rates were monitored every day until day 14, at which point the remaining animals were euthanized. FAAH inhibition was successful in lowering mortality, as depicted in Figure 1A. In a similar manner, the delivery of URB878 daily greatly lessens the body weight loss brought on by bleomycin, seen in Figure 1B. Moreover, administration of bleomycin induced significant alterations in the lung architecture, as highlighted in alveolar thickening, accumulation of leukocytes, and increased extracellular matrix and fibroblasts (Figure 1D and Aschroft score Figure 1F) compared to the sham group (Figure 1C and Aschroft score Figure 1F). Daily administration of URB878 at the dose of 5 mg/kg for 14 days significantly decreased the histological damage that bleomycin induced (Figure 1E and Aschroft score Figure 1F). Additionally, the increase in the wet:dry lung weight ratio due to infiltration of inflammatory cells and edema found in the bleomycin group where significantly reduced after URB878 treatment (Figure 1G).

### 2.2. URB878 Administration Reduced Bleomycin-Induced Inflammatory Cell Migration

**Figure 2:**
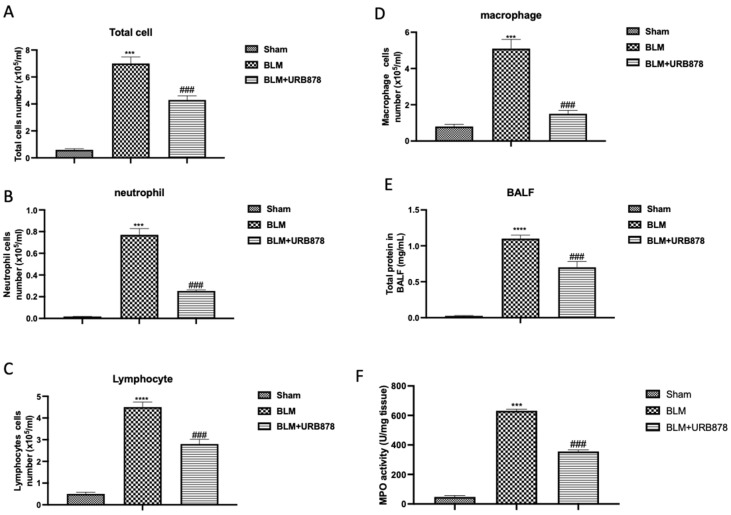
URB878 decreased bleomycin-induced inflammatory cell migration. Total (**A**) and differential cell counts (**B**) for neutrophils; (**C**) for lymphocytes; (**D**) for macrophages in bronchoalveolar lavage fluid (BALF); total protein concentration in BALF (**E**), and MPO activity (**F**). Data are expressed as the mean ± SEM of n = 6 animals for each group. *** *p* < 0.001 vs. sham; **** *p* < 0.0001 vs sham; ### *p* < 0.001 vs. bleomycin.

The endothelial lining of capillaries and small arteries was damaged by bleomycin injury, which is characterized by vascular congestion and increased microvascular permeability, both of which cause an inflammatory response [32]. In comparison to the sham group, we observed an increase in protein content (Figure 2E) and cellular density in BAL following bleomycin injection (Figure 2A–D for total cells, neutrophils, lymphocytes, and macrophages, respectively). A increase in neutrophilic migration was also reflected in the increase in MPO activity, a well-known marker. As shown in Figure 2F, MPO activity significantly increased after bleomycin administration compared to the sham group. After 14 days of oral administration of URB878 at the dose of 5 mg/kg, we found a significant decrease in all the parameters evaluated.

### 2.3. URB878 Administration Reduced Cell Infiltration

**Figure 3:**
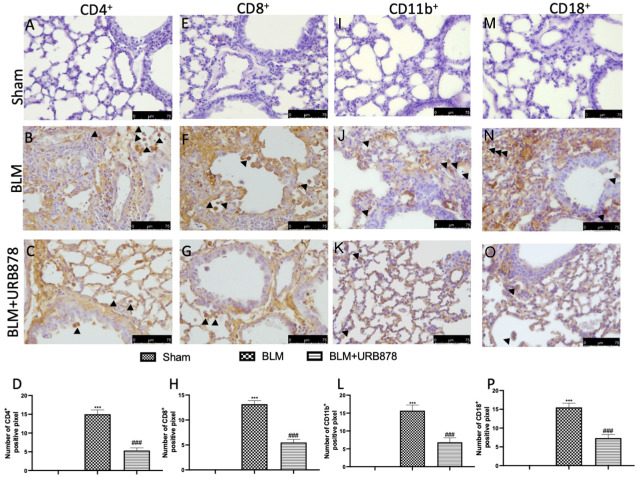
URB878 administration reduced cell infiltration. Sham (**A**); BLM (**B**); BLM+URB878 5 mg/kg (**C**); graphical analysis (**D**) for CD4. Sham (**E**); BLM (**F**); BLM+URB878 5 mg/kg (**G**); graphical analysis (**H**) for CD8. Sham (**I**); BLM (**J**); BLM+URB878 5 mg/kg (**K**); graphical analysis (**L**) for CD11b. Sham (**M**); BLM (**N**); BLM+URB878 5 mg/kg (**O**); graphical analysis (**P**) for CD18. Values are the means ± SEM of 6 mice for all groups. Photos shown are representative of the results obtained. See manuscript for further details. *** *p* < 0.001 vs. sham; ### *p* < 0.001 vs. bleomycin. Arrows indicate positive cells.

CD8+ and CD4+ T-cells play a key role in BLM-induced fibrosis, because a reduction of individual T-cell subsets attenuates lung fibrosis, and fibrosis is completely prevented by simultaneous depletion of both T-cell subsets [33]. Additionally, CD11b and CD19 signaling is associated with the development of pulmonary fibrosis by controlling B-cell infiltration during the fibrotic phase of the response to bleomycin [34,35]. Bleomycin-treated mouse lung sections exhibited positive CD4 (Figure 3B and densitometric analysis Figure 3D), CD8 (Figure 3F and densitometric analysis Figure 3H), C11b (Figure 3J and densitometric analysis Figure 3L), and CD18 (Figure 3N and densitometric analysis Figure 3P) staining, primarily in the interstitial inflammatory cell infiltration and the alveolar pneumocyte layer. In contrast, it was discovered that mice treated with FAAH inhibitor had less CD4 (Figure 3C and densitometric analysis Figure 3D), CD8 (Figure 3G and densitometric analysis Figure 3H), C11b (Figure 3K and densitometric analysis Figure 3L), and CD18 (Figure 3O and densitometric analysis Figure 3P). No positive staining was found in the sham animals (Figure 3A,E,I and Figure 3M and densitometric analysis Figure 3D,H,L and Figure 3P, respectively).

### 2.4. URB878 Decreased Bleomycin-Induced Fibrosis

**Figure 4:**
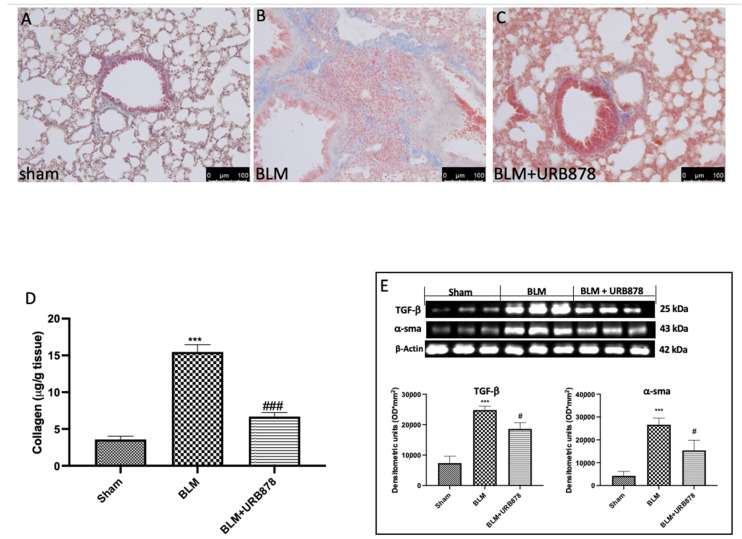
URB878 decreased bleomycin-induced fibrosis. (**A**) Sham, (**B**) bleomycin, and (**C**) bleomycin+URB878 5 mg/kg; (**D**) soluble collagen; (**E**) Western blot and relative densitometric analysis of α-SMA and TGF-β. The photos are demonstrative of at least three experiments carried out on different experimental days. The data are expressed as the mean ± SEM of n = 6 animals for each group. *** *p* < 0.001 vs. sham; # *p* < 0.05 vs. bleomycin; ### *p* < 0.001 vs. bleomycin.

To assess the degree of pulmonary fibrosis in lung tissue, Masson’s trichrome staining, Western blot analysis of α-SMA and TGF-β were utilized. In comparison to the sham group, we observed a significant increase in fibrotic lesions and collagen buildup in the lungs of mice following bleomycin injection (Figure 4B for Masson Staining and Figure 4D for soluble collagen). Contrarily, collagen deposition and fibrotic scars were dramatically reduced when URB878 was given daily at a dose of 5 mg/kg. The expressions of α-SMA and TGF-β are another well-established indicator of fibrosis in the literature. [36,37]. As supposed, we found a significant increase in the group subjected to bleomycin when compared to the sham group (Figure 4E). Significant decreases in α-SMA and TGF-β expressions were found after URB878 administration.

### 2.5. URB878 Reduced Mast Cells Degranulation

**Figure 5:**
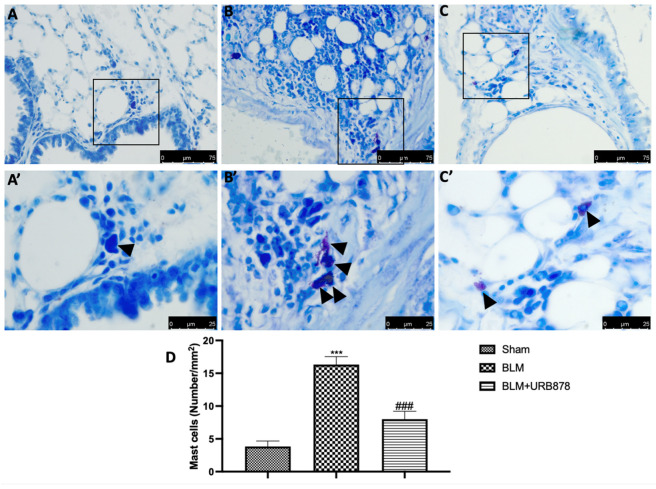
URB878 reduced mast cell degranulation. (**A**,**A’**) Sham, (**B**,**B’**) bleomycin, and (**C**,**C’**) bleomycin+URB878 5 mg/kg; (**D**) mast cell count. The photos are demonstrative of at least three experiments carried out on different experimental days. The data are expressed as the mean ± SEM of n = 6 animals for each group. *** *p* < 0.001 vs. sham; ### *p* < 0.001 vs. bleomycin. Arrows indicates mast cells.

Most tissues, especially those exposed to the outside world (such the airways), experience local mast cell maturation. Due to this, MCs are probably among the first immune cells exposed to proinflammatory and toxic substances, along with dendritic cells and macrophages. In addition, it has been noted that patients with various types of lung fibrosis have large numbers of MCs in their lungs [38]. In order to assess mast cell activation and recruitment in the inflamed tissues, toluidine blue staining was performed on the lung tissues from mice that had received bleomycin injections. Mast cells were not found in the tissues of animals that had received sham treatment (Figure 5A,A’), whereas tissues from animals that had received vehicle treatment showed more infiltrating mast cells (Figure 5B,B’). URB878 at the dose of 5 mg/kg (Figure 5C,C’) reduced the infiltration and the degranulation in the lungs (Figure 4D).

### 2.6. URB878 Administration Decreased Bleomycin-Induced Inflammation

**Figure 6:**
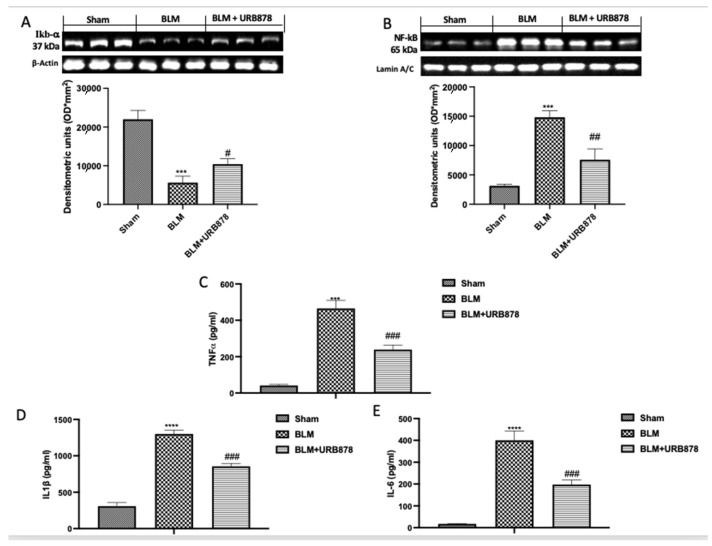
URB878 administration decreased bleomycin-induced inflammation. Western blots and relative densitometric analysis for IkB-α (**A**); NF-kb (**B**); ELISA quantification for TNF-α (**C**), IL-1β (**D**), and IL-6 (**E**). The data are expressed as the mean ± SEM of n = 6 mice/group. *** *p* < 0.001 vs. sham; **** *p* < 0.0001 vs. sham; # *p* < 0.05 vs. bleomycin; ## *p* < 0.01 vs. bleomycin; ### *p* < 0.001 vs. bleomycin.

Numerous studies have shown that BLM can activate the NF-B signaling pathway, which is crucial for regulating inflammation. Additionally, it plays a significant part in lung fibrosis brought on by BLM [39]. To investigate the molecular pathway involved, we examined the activation of NF-kb pathway by Western blot. As supposed, we found a significant decrease in IkB-α expression (Figure 6A), and consequently a significant increase in NF-kb translocation (Figure 6B) in the bleomycin group compared to the sham group. After the treatment with URB878, we found significantly restored IkB-α expression (Figure 6A), and a significant decrease in NF-kb translocation (Figure 6B). The same trend was observed in the analysis of TNF-α (Figure 6C), IL-1β (Figure 6D), and IL-6 (Figure 6E).

### 2.7. URB878 Administration Reduced Nitrosative Stress and DNA Damage

**Figure 7:**
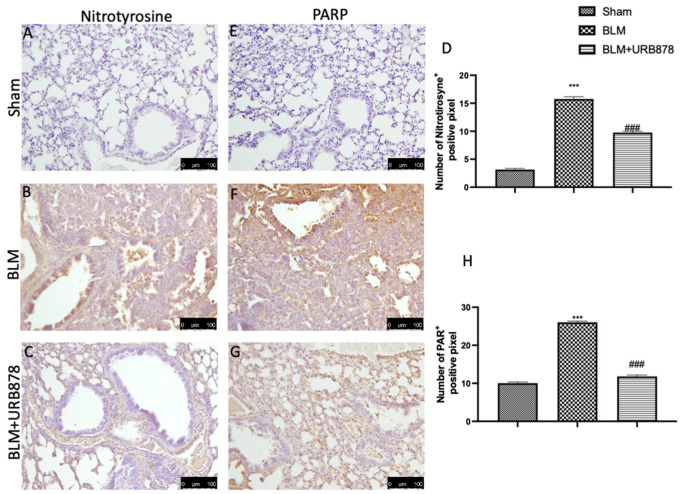
URB878 reduced nitrosative stress and DNA damage. Sham (**A**); BLM (**B**); BLM+URB878 5 mg/kg (**C**); graphical analysis (**D**) for nitrotyrosine. Sham (**E**); BLM (**F**); BLM+URB878 5 mg/kg (**G**); graphical analysis (**H**) for PARP. Values are the means ± SEM of 6 mice for all group. The photos shown are representative of the results obtained. See manuscript for further details. *** *p* < 0.001 vs. sham; ### *p* < 0.001 vs. bleomycin.

After bleomycin administration, nitrotyrosine and PARP immunoreactivity were frequently seen to rise in the lung [40]. As compared to the control group (Figure 7A,E, see Figure 7D and Figure 7H, respectively), the lung tissue from the bleomycin-treated group had a higher percentage of nitrotyrosine- and PARP-positive cells, according to an immunohistochemical examination (Figure 7B and Figure 7F, respectively). Nitrotyrosine and PARP expression were also considerably reduced after receiving URB878 at a dose of 5 mg/kg (Figure 7C and Figure 7H, respectively; see graphs D and H).

### 2.8. URB878 Reduced Adhesion Molecule Expression That Bleomycin Induced

**Figure 8:**
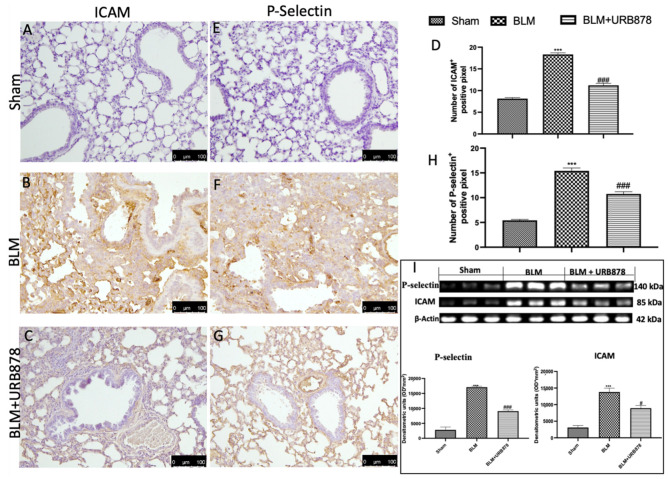
URB878 administration decreased ICAM and P-selectin expression. Sham (**A**); BLM (**B**); BLM+URB878 5 mg/kg (**C**); graphical analysis (**D**) for ICAM. Sham (**E**); BLM (**F**); BLM+URB878 5 mg/kg (**G**); graphical analysis (**H**) for P-selectin; Western blots for ICAM and P-selectin and relative densitometric analysis (**I**). Values are the means ± SEM of 6 mice for all group. Photos shown are representative of the results obtained. See manuscript for further details. *** *p* < 0.001 vs. sham; # *p* < 0.05 vs. bleomycin; ### *p* < 0.001 vs. bleomycin.

ICAM and P-selectin staining intensity considerably rose in lung tissue slices from the bleomycin-treated group as compared to the control group (Figure 8B and Figure 8F, respectively; see graphs D and H). Both stains were greatly lessened by URB878 at a dose of 5 mg/kg (see graphs D and H, respectively, in Figure 8C,H). Additionally, we examined the expression of adhesion molecules using Western blots and discovered the same pattern as immunohistochemistry (Figure 8I).

## 3. Discussion

No one has examined the function of FAAH in an experimental model of lung injury caused by bleomycin up until this point, despite the research conducted using FAAH inhibitors or with genetically modified animals. Histologically, the acute phase of ALI is marked by inflammatory cell infiltration and rupture of the alveolar–capillary barrier, which results in a proteinaceous exudate that floods the alveolar spaces, inhibits gas exchange, and precipitates respiratory failure [41,42]. In our study, we found that by inhibiting the FAAH enzyme, the entire histological damage decreases. The interstitium fibrosis, collagen, elastic, and smooth muscle elements; architectural remodeling, and chronic interstitial inflammation with variable increases in lymphocytes, neutrophils, plasma cells, macrophages, eosinophils, and mast cells; CD4-, CD8-, CD11b-, and CD18-positive cells; hyperplasia of type II cells; and hyperplasia of endothelial cells are common pathologic features in IPF. In our study, we found that after the daily administration of URB878 at the dose of 5 mg/kg, fibrosis, as well as cellular migration in BALF, and mast cell degranulation were significantly inhibited. Moreover, these inflammatory cells that migrate to and proliferate in sites of damage produce many cytokines through the activation of the NF-κB pathway leading to the production of fibroblasts [39]. In our study, FAAH inhibition by URB878 at the dose of 5 mg/kg significantly decreased NF-kb translocation and as a consequence, the release of TNF-α, IL-1β, and IL-6. Nitric oxide (NO), a messenger molecule with intricate biological functions, is another chemical messenger linked to oxidative stress. It reacts with ROS to produce highly reactive nitrogen intermediates. Many cell types, including inflammatory cells, alveolar and bronchiolar epithelia, vascular endothelia, alveolar macrophages, neutrophils, and mast cells, all contribute to the production of NO in the lungs [43]. We discovered a considerable rise in nitrotyrosine expression in our investigation, which is in accordance with the literature. This rise was lessened by FAAH inhibition, which returned expressions to normal levels. Additionally, we assessed the DNA damage brought on by the administration of bleomycin and discovered that FAAH inhibition by URB878 at a dose of 5 mg/kg was capable of lowering PARP activation. By encouraging leukocyte adherence to vascular wall endothelium, P-selectin and intercellular adhesion molecule-1 (ICAM-1) engage in inflammatory processes associated with BLM [44]. FAAH inhibition significantly reduces the expression of both molecules.

## 4. Materials and Methods

### 4.1. Animals

CD1 mice (25–30 g, Envigo, Milan, Italy) were employed. The University of Messina Review Board for animal care (OPBA) approved the study. All animal experiments agreed with the new Italian regulations (D.Lgs 2014/26), EU regulations (EU Directive 2010/63), and the ARRIVE guidelines.

### 4.2. Experimental Design and Groups

Bleomycin administration was performed as previously described [45,46,47]. In detail, a single intratracheal injection of bleomycin sulphate (1 mg/kg body weight) was administered to mice. To ensure delivery to the distal airways, 100 μL of fluid was injected at the end of the exhalation. There was 300 mL of air released right after that [37,48,49,50,51] (See Supplementary Figure S1).

Mice were randomly divided into groups:

(I) Bleomycin: animals that receive one injection of bleomycin at time 0.
(II) Bleomycin+URB878: mice were subjected to the bleomycin injection described above and treated orally with URB878 at the dose of 5 mg/kg dissolved in a vehicle consisting of 10% PEG-400, 10% Tween-80, and 80% saline for the first time 1 h after bleomycin injection and once a day for 14 days.
(III) Sham: animals that were exposed to the vehicle.
(IV) Sham groups+URB878: animals received URB878 dissolved in a vehicle for 14 days at the dose of 5 mg/kg.

Doses were chosen based on a work carried out in our lab in an experimental model of carrageenan-induced acute lung injury [52].

At the end of experiment, mice were euthanized, and lung tissue and bronchoalveolar lavage fluid (BALF) were collected as previously described [49,53,54,55,56,57].

### 4.3. Measurement of Lung Edema

The wet lung weights were recorded at the conclusion of the experiment. The lungs were then dried for 48 h at 180 °C before being weighed once more. The ratio of the tissue’s wet to dry weight was used to determine the water content of the lungs [58,59].

### 4.4. Histopathological Evaluation with Hematoxylin/Eosin, Toluidine Blue, and Masson

Lungs were dehydrated, embedded in paraffin, and cut at 7 microns. Slices from each group were stained with hematoxylin/eosin (H/E), toluidine blue, or Masson trichrome, examined under a light microscope with a Leica DM6 attached to an imaging system (LasX Navigator), and graded by two researchers who were blind to the experimental groups. According to Ashcroft et al.’s method, the degree of lung fibrosis and damage was evaluated [37,45,46,47,48,58,59].

### 4.5. Bronchoalveolar Lavage (BAL)

Mice were euthanized at the conclusion of the experiment, and the tracheas were cannulated to carry out the lavage for cell counting, as previously described [58,59]. Additionally, from BAL, we analyzed the total protein content using a DC Protein Assay kit (Bio-Rad Laboratories, Hercules, CA, USA) as previously described [60,61].

### 4.6. Western Blot Analysis of Cytosolic and Nuclear Extracts

Extracts of the cytosol and nucleus were prepared, as previously mentioned [62,63,64,65,66]. The following primary antibodies were used: anti-α-sma antibody (1:250, Santa Cruz Biotechnology (SCB), Dallas, TX, USA), anti-Iκbα (1:500, SCB, #sc-1643), anti-ICAM (1:500, SCB), anti-pselectin (1:500, SCB), anti-TGFβ (1:500, SCB) and anti-nfκb (1:500, SCB, #sc8414) in 1× PBS, 5% *w*/*v* non-fat dried milk, and 0.1% Tween 20, at 4 °C overnight [67,68,69]. Western blots were further investigated for the cytosolic fraction using an anti-β-actin protein antibody (1:500, SCB, Dallas, TX, USA). For nuclear fraction with lamin A/C (1:500, Sigma-Aldrich Corp., Milan, Italy), the same techniques were applied [70,71]. According to the manufacturer’s instructions, an enhanced chemiluminescence (ECL) detection system reagent (Thermo, Monza, Italy) was used to examine the signals. Using densitometry and the BIORAD Chemi-DocTM XRS+ software (Bio-Rad, Milan, Italy), the relative expression of the protein bands was measured [72,73].

### 4.7. Cytokine Measurement

Using ELISA kits from R&D Systems in Minneapolis, Minnesota, USA, we assessed the exudates’ TNF-α or IL-6 or IL-1β concentrations. The results are expressed as pg/mL [74,75,76,77,78,79,80,81].

### 4.8. Immunohistochemical Localization of Nitrotyrosine, Poly(ADP-Ribose), CD4, CD8, CD11b, CD18, ICAM, and P-Selectin

At the end of the experiments, slices of lung tissue were incubated with anti-ICAM-1 (1/100 in PBS, SCB), anti-P-selectin (1/100 in PBS, SCB), anti-CD-8 (1:450 in PBS, sc-7970), anti-CD-11b (1:450 in PBS, sc-1186), anti-CD18 (1:350 in PBS, sc-18862), anti-CD4 (1:350 in PBS, sc-13573), anti-PAR (1/100 in PBS, SCB), and anti-nitrotyrosine (1:200 in PBS Millipore) as previously described [64,69,82,83,84]. After that, sections were treated with peroxidase-conjugated goat anti-rabbit IgG or bovine anti-mouse IgG secondary antibodies (1:2000 Jackson Immuno Research, West Grove, PA, USA). A biotin-conjugated goat anti-rabbit IgG or the biotin-conjugated goat anti-mouse IgG and avidin-biotin peroxidase combination (Vector Laboratories, Burlingame, CA, USA) were used to identify specific markers. Using an imaging device (Leica DM6, Milan, Italy), immunohistochemical pictures were captured (LasX Navigator, Milan, Italy). The number of positive pixels were analyzed with Image J plug-in [64,69,82,83,84]. All immunohistochemical analyses were carried out by two observers blind to the treatment [67,68,85,86,87].

### 4.9. Soluble Collagen Assay

The total content of lung collagen was measured using the manufacturer’s recommendations using the Sircol Soluble Collagen Assay (Biocolor, Newtownabbey, Northern Ireland), a modification of the Sirius red technique [37,58].

### 4.10. Survival Rate

Mortality was assessed daily up to day 14 and expressed as percental survival.

### 4.11. Mieloperoxidase (MPO) Evaluation

Mieloperoxidase (MPO) activity was measured after BLEO injection for 14 days in the same way as originally described. Briefly, MPO activity was determined to be the amount of enzyme that oxidizes 1 μmol of peroxide per minute at 37 °C, and it was measured in units per gram of wet tissue weight [88].

### 4.12. Materials

Unless otherwise stated, all compounds were purchased from Sigma-Aldrich.

### 4.13. Synthesis of URB878

FAAH inhibitor URB878 was synthesized as previously reported [29,52].

### 4.14. Statistical Evaluation

The data in this study are presented as the average ± SEM and represent at least three experiments conducted on various days. N stands for the number of animals used in in vivo experiments, as determined using G*Power 3.1 software (Die Heinrich-Heine-Universität Düsseldorf, Düsseldorf, Germany). An expert histopathologist conducted the data analysis, and the outcomes were investigated using a one-way ANOVA and a Bonferroni post hoc test for multiple comparisons. A *p*-value of 0.05 or less was regarded as significant.

## 5. Conclusions

Our data clearly demonstrate for the first time that the inhibition of FAAH activity was able to counteract not only the bleomycin-induced histological alteration but also the cascade of related inflammatory events. These processes might be the cause of the decrease in neutrophil migration and nitrosative stress that occurs during the inflammatory response. The combined results of this research strengthen our understanding of how FAAH modulates inflammation pathophysiologically and lends credence to its therapeutic potential for chronic lung illnesses. We suggest that it acts by enhancing anandamide levels through the inhibition of its hydrolysis, and that these were able to modulate inflammation, and by down-modulating mast cell degranulation and activation of one or more members of the peroxisome proliferator-activated receptor (PPARs) family of nuclear receptors and/or a cannabinoid CB2-like receptor, decreasing inflammation and nitrosative stress. Further research is needed to better investigate how and what protein channels are involved in IPF.

## Data Availability

The data used to support the findings of this study are available from the corresponding author upon request.

## Supplementary Materials

The following supporting information can be downloaded at: https://www.mdpi.com/article/10.3390/ijms241210125/s1.
