# Supplementary figures and images for: Inhibition of Fatty Acid Amide Hydrolase (FAAH) Regulates NF-kb Pathways Reducing Bleomycin-Induced Chronic Lung Inflammation and Pulmonary Fibrosis
Source: Int J Mol Sci. 2023 Jun 14;24(12):10125. doi: 10.3390/ijms241210125 (PMC10298572; doi:10.3390/ijms241210125)

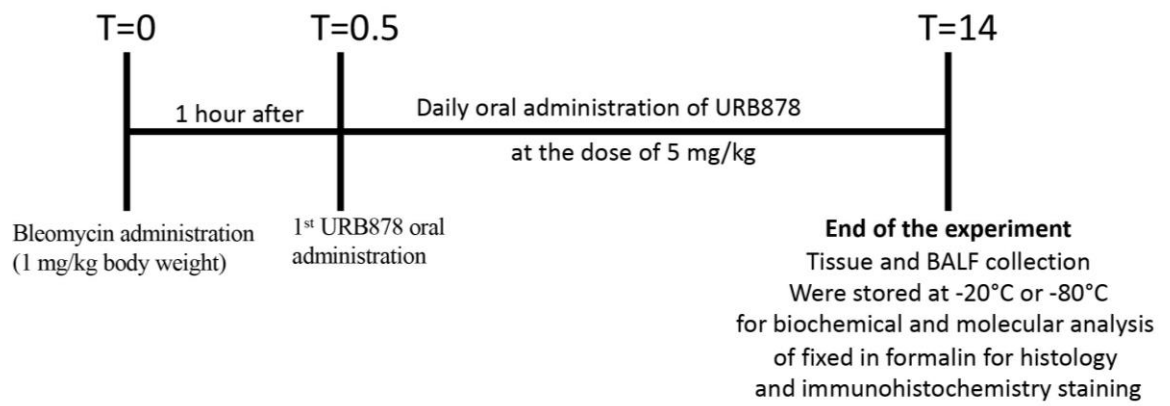

Figure S1. Experimental design of bleomycin induced chronic lung injury.

Supplement: Supplementary file 1 [file ijms-24-10125-s001.zip › ijms-2394862-supplementary.pdf]
